# Jaw tumor in recurrent primary hyperparathyroidism: A case report

**DOI:** 10.1016/j.ijscr.2018.09.035

**Published:** 2018-10-04

**Authors:** Farzaneh Amini Nezhad, Moloud Payab, Sara Nayebandi, Shirin Hasani-Ranjbar

**Affiliations:** a Endocrinology and Metabolism Research Center, Endocrinology and Metabolism Clinical Sciences Institute, Tehran University of Medical Sciences, Tehran, Iran; b Obesity and Eating Habits Research Center, Endocrinology and Metabolism Molecular -Cellular Sciences Institute, Tehran University of Medical Sciences, Tehran, Iran; c Department of Radiology, Shariati Hospital, Tehran University of Medical Sciences, Tehran, Iran

**Keywords:** Brown tumor, Hyperparathyroidism, Hypercalcemia, Hyperparathyroidism jaw-tumor syndrome

## Abstract

**Background:**

Brown tumor may present as uni/multilocular osteolytic lesions with bone expansion, bone pain or pathologic fracture in primary, secondary and tertiary hyperparathyroidism. However, recently, such presentation is rare due to early detection using blood screening techniques before symptomatic bone lesions appear.

**Case Report:**

A 65-year-old woman was admitted to our hospital with generalized bone pain and a progressive painless mass in her jaw appeared since 6 months ago. She had a history of two times parathyroidectomy. In recent hospitalization, she presented with the complaint of weakness, bone pain and a progressive swelling in her jaw. Laboratory analysis showed a hypercalcemia and plasma PTH of 398 pg/ml. Dual energy X-ray (DXA) showed osteoporosis at the neck of hip and lumbar spine. The neck MRI revealed an 11 * 6 mm soft tissue nodule posterior to left thyroid lobe and a 20*18 mm T2 hypersignal, T1hyposignal nodule at right thyroid lobe. The SPECT-CT scintigraphy with 99mTc-MIBI was suggestive of bilateral parathyroid adenomas and/or parathyroid hyperplasia and showed a MIBI- avid lytic lesion in the mandible. Surgical management was performed. After removing the adenoma the PTH marked a decrease from the initial value to 57 pg/ml. The histological examination revealed the thyroid tissue with MNG and a left parathyroid adenoma. Follow-up after surgery revealed normal blood calcium and urine calcium levels with no increase. PTH levels also did not increase. The patient was treated with calcium and vitamin D supplements and the jaw mass decreased gradually.

**Conclusions:**

Primary or secondary HPT may be recognized by the presence of an osteolytic lesion with giant cells, a condition referred as brown tumor. Consideration should be made that non-specificity of hypercalcemia symptoms can also be a bone tumor including jaw lesion as a manifestation of hyperparathyroidism.

## Highlights

• Brown tumor may present as uni/multilocular osteolytic lesions with bone expansion, bone pain or pathologic fracture in primary, secondary and tertiary hyperparathyroidism. Recently, such presentation is rare because of early detection before symptomatic bone lesions appear due to improved blood screening techniques.
• The reported case is a 65-year-old female presented with recurrent hyperparathyroidism and a tumoral mass in her jaw.
• Although recurrent Hypercalcemia with high serum PTH level, jaw tumor, and bilateral parathyroid adenomas in99mTc-MIBI parathyroid scintigraphy suggesting HPT-JT, her old age, lack of similar familial history, absence of nonendocrine malignancy as well as evidence of malignancy in the patient's parathyroid pathology, this diagnosis was not supported.

## 1 Introduction

Primary hyperparathyroidism (PHPT) is a common endocrinopathy that mainly affects women in the fifth decade of life. It may be caused by a single adenoma (75%–85%), hyperplasia (10%–20%), multiple adenomas (4%–5%) or carcinoma (1% of the cases).

Less than 10% of PHPT is due to genetic disorders. It is generally detected in younger ages, affects both genders equally, and has a high recurrence rate and may be associated with the positive family history, multiple endocrinopathies, multiglandular parathyroid disease (hyperplasia, multiple adenomas) or carcinoma. Genetic syndromes causing PHPT include multiple endocrine neoplasia (MEN type 1 or 2 A), familial hypocalciuric hypercalcemia (FHH), and hyperparathyroidism jaw-tumor syndrome [1,2].

Primary hyperparathyroidism was previously characterized by severe hypercalcemia, recurrent nephrolithiasis and reduced renal function, osteoporosis and osteitis fibrosa cystica. However, in the 1970s, mild hypercalcemia became easily detectable so that the routine use of serum calcium, as part of a biochemical screening profile, became a common practice. most people are asymptomatic at the time of diagnosis [[3], [4], [5]].

Osteitis fibrosa cystica is the skeletal manifestation of primary hyperparathyroidism which is manifested by salt-and-pepper degranulation of the skull, tapering of the distal clavicle, subperiosteal resorption of the distal phalanges, bone cysts, and brown tumors [6].

Brown tumor may present as uni/multilocular osteolytic lesions with bone expansion, bone pain or pathologic fracture. Recently, such presentation is rare due to early detection as a result of the improved blood screening techniques before symptomatic bone lesions appear [[7], [8], [9]].

The only cure for primary hyperparathyroidism is surgical removal of the parathyroid adenoma or adenomas. Persistent and recurrent PHPT after surgery may occur when an incident parathyroid adenoma cannot be located. an inadequate resection is performed in a patient with multigland hyperplasia, an initial adequate resection was performed in the setting of familial disease, but recurrent disease develops in residual gland(s) that were “normal” at the time of initial exploration; a second occult parathyroid adenoma is left *in situ*; or residual hyperfunctioning parathyroid tissue remains in rare cases of parathyromatosis or parathyroid carcinoma. The most common cause is surgeon inexperience in locating and adequately excising a parathyroid adenoma [10].

This is a report of a 65-year-old case of PHPT with recurrent hyperparathyroidsm and a tumoral mass in her mandible. This work has been reported in line with the SCARE criteria [11].

## 2 Case report

A 65-year-old woman was admitted to our hospital with generalized bone pain and a progressive painless mass in her jaw since 6 months ago. She had a history of two times parathyroidectomy, first being in 2008 (10 years ago). The patient was evaluated for weakness and elevated serum level of Ca and PTH in laboratory data, the parathyroid scintigraphy with 99mTc-MIBI revealed a left parathyroid adenoma. Then, she underwent surgery and the pathological findings was in favor of left parathyroid adenoma.

During the follow up in 2010 (8 years ago) she had been hospitalized due to the elevated serum level of Ca and PTH again. The neck ultrasonography revealed a multi nodular thyroid goiter. The parathyroid scintigraphy was performed which showed bilateral parathyroid hyperplasia. The patient subsequently underwent a second neck operation with removal of right parathyroid glands and exploration of left side of neck. However, the left parathyroid glands were not found. The pathology report was a thyroid nodule, parathyroid tissue with hyperplastic changes.

**Fig. 1:**
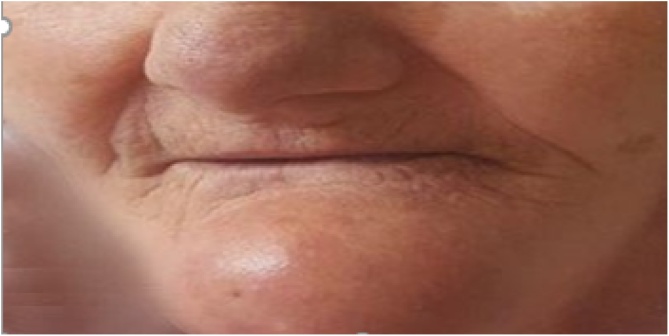
a well-circumscribed 5 cm × 5 cm swelling in the mandible.

In her recent hospitalization, she presented with complaint of weakness, bone pain and a progressive swelling in her jaw (Fig. 1). On examination a well-circumscribed firm and non-tender swelling in the mandile measuring about 50 mm × 50 mm was revealed. The surface skin over it was shiny but there was no ulcer or discharge. Laboratory analysis showed a hypercalcemia: 13.4 mg/dl (normal range: 8.6–10.4) and plasma PTH was 398 pg/ml (normal range: 8–76 pg/ml), 24-hour urinary calcium was at 328 mg/24 h (N < 300). The vitamin D level was subnormal at 35 nmol/lit (normal range: 45–144 nmol/lit) and dual energy X-ray (DXA) showed osteoporosis with a T-score of -3.8 at the neck of hip and -3.7 at lumbar spine.

She had a pathology report of the mandible tumor biopsy that had been done recently in another center via oral cavity which was suggestive of a central giant cell granuloma.

The initial management with rehydration, parentral bisphosphonate and diuretics was done and then preoperative localization investigations were performed.

**Fig. 2:**
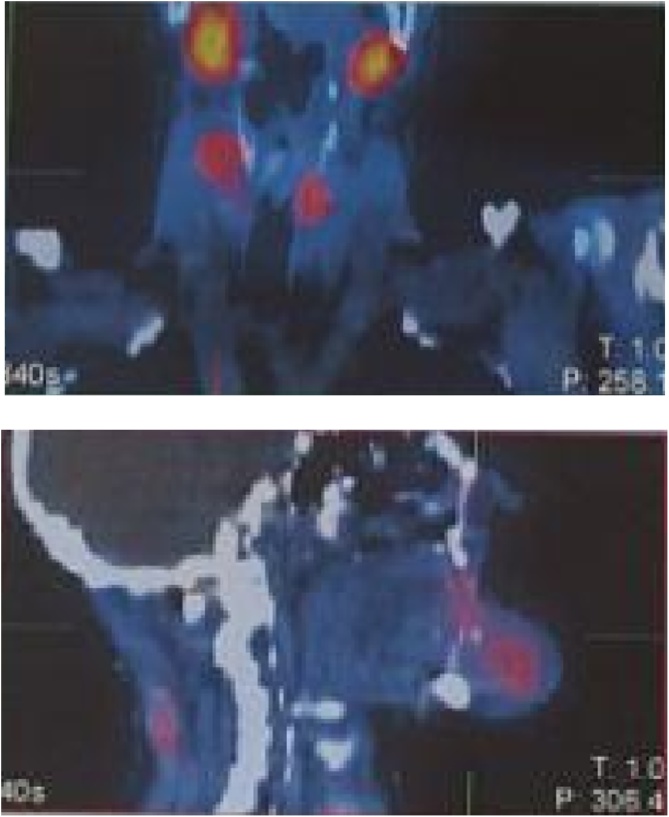
SPECT-CT parathyroid scan. bilaretal parathyroid adenomes and parathyroid hyperplasia.

The SPECT-CT scintigraphy with 99mTc-MIBI was suggestive of bilateral parathyroid adenomas and/or parathyroid hyperplasia and showed a MIBI- avid lytic lesion in the mandible (Fig. 2).

**Fig. 3:**
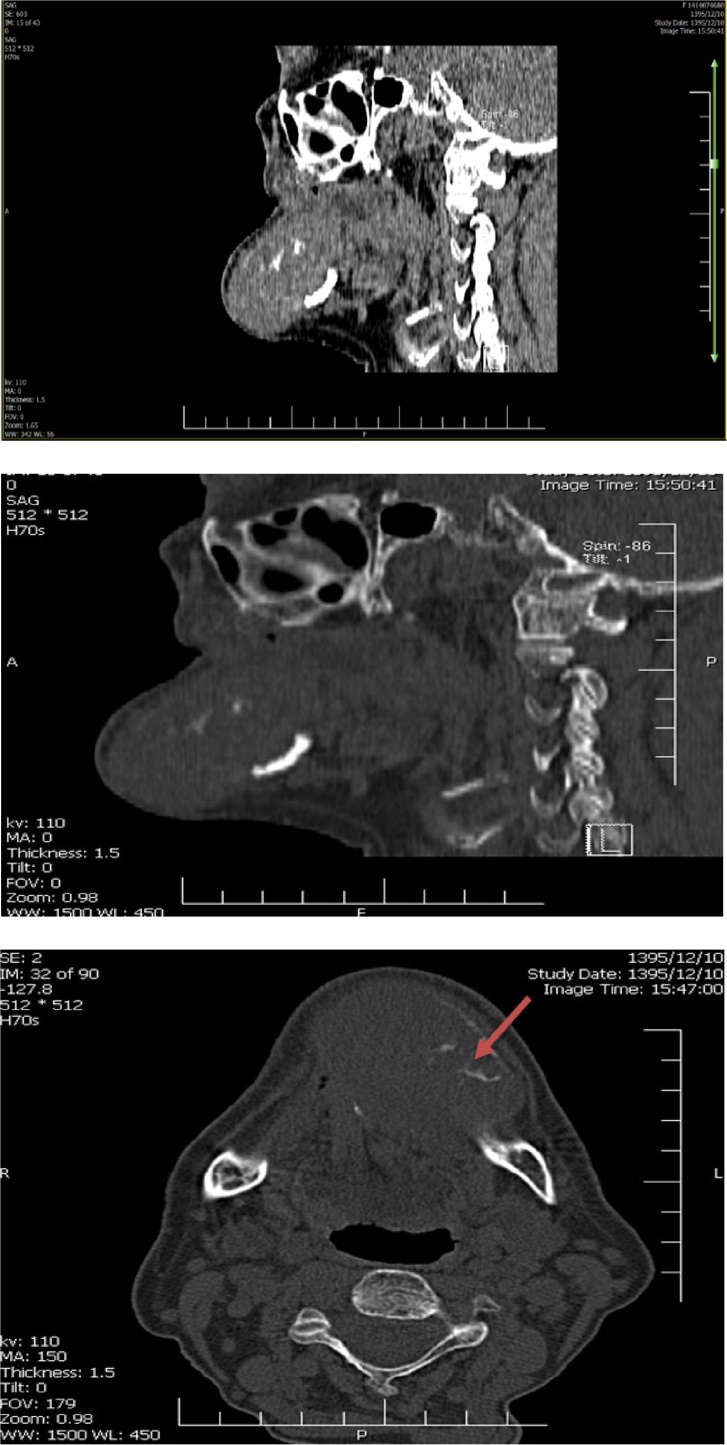
Spiral mandible CT scan without contrast: A well-defined, lytic expansle lesion in mandibular body (arrow).

In the spiral mandible CT scan without contrast there was a 53 × 43 × 39 mm expansile lytic mass lesion at mandibular symphysis and left side of mandibular body, which showed well defined nonsclerotic margin without cortical disruption. No periosteal reaction was evident and bony trabecular remnant was sited in central part. Mandibular cortex has been involves by mentioned mass at both sides and extension of mass posteriorly to oral cavity at sublingual space and anteriorly to subcutaneous region was depicted (figure 3).

Surgical management consisted of total thyroidectomy and total parathyroidectomy with autotransplantation of about 50 miligrams of excised parathyroid tissue was performed. After removing the adenoma, the PTH marked a decrease from initial (398 pg/ml) value to 57 pg/ml.

The histological examination revealed the thyroid tissue with MNG and a left parathyroid adenoma. Follow-up after surgery revealed normal serum calcium and urine calcium levels with no increase. PTH levels also did not increase. The patient was treated with calcium and vitamin D supplements and the jaw mass gradually decreased.

## 3 Discussion

Brown tumor or osteoclastma is a bony lytic lesion which may be presented in primary, secondary or tertiary hyperparathyroidism. The name implies the color of the lesion, caused by the hemorrhagic vascularity and hemosiderin sedimentation.

Histologically, it is a giant cell lesion that can occur in trabecular bones of jaw, ribs, and phalanx bone, and is associated with demineralization and replacement of bone tissue with connective one. Currently, due to the diagnostic and screening methods, this manifestation has occurred rarely and is regressed by hyperparathyroidism therapies which are better corrected by Parathyroidectomy [12].

Rarely, hyperparathyroidism can occur in patient with hyperparathyroidism-jaw tumor syndrome (HPT-JT), which is a rare Automosal Dominant disorder characterized by parathyroid adenoma or carcinoma and jaw fibro-osseous tumor. Other manifestations can include kidney wilms tumor, beningn cycts, and papillary thyroid carcinoma. This syndrome is caused by the CDC73 inactivation mutation on the chromosome 1q25 responsible for protein excretion by a tumor suppression function, called parafibromin [13].

Hyperparathyroidism in these patients occurs in younger ages contrary to sporadic forms, also parathyroid adenomas are usually single in them, but may be multiple or likely to get recurrences [14].

Ossifying fibroma (OF) is a benign neoplasm in the craniofacial region which predominantly affects the mandible and is more common in women [12]. The OF lesion discrimination from the Brown tumor can be either difficult or impossible for clinical and radiological perspectives, and could be diagnosed only with no improvement after hyperparathyroidism correction and the absence of giant cell lesions in the pathology [14]. OF treatment includes complete surgical excision of bone tumor and grafting enucleated lesion [15].

The reported case is a 65-year-old female presented with recurrent hyperparathyroidism and a tumoral mass in her jaw. The patient underwent parathyroid surgery through which the histology of the lesion confirmed the parathyroid adenoma without evidence of malignancy. After surgery, hypercalcemia was corrected and the jaw tumor was regressed gradually.

Although recurrent Hypercalcemia with high serum PTH level, jaw tumor, and bilateral parathyroid adenomas in99mTc- MIBI parathyroid scintigraphy was suggestive of HPT-JT, but due to the old age of patient, lack of similar familial history, absence of nonendocrine malignancy as well as no evidence of malignancy in the patient's parathyroid pathology, this diagnosis was not supported. Also, the features of her jaw mass, including the lytic lesion in radiology, the presence of giant cell in the maxillary mass pathology and gradual improvement following parathyroidectomy were not in favor of diagnosing ossifying fibroma.

Currently osteitis fibrosa cystica (brown tumor) is rarely diagnosed as a clinical presentation of primary hyperparathyroidism and the clinical and radiological examination of other areas with common involvement in hyperparathyroidism including ribs and long bones (e.g. femur, tibia, and pelvis) revealed no evidence of similar lesion other than mandibular lesion. However, history of hyperparathyroidism, characteristics of the jaw lesion, presence of a giant cell in the histology of the lesion, as well as gradual regression of tumor after surgical intervention revealed a very rare clinical manifestation of primary hyperparathyroidism in a patient with a single mandibular brown tumor and nonspecific symptoms of hypercalcemia (Fig. 3).

## Conflicts of interest

The authors have declared that they have no competing interests.

## Funding source

This study was sponsored by Tehran University of Medical Sciences (Endocrinology and Metabolism Research Institute).

## Ethical approval

Ethical approval has been exempted by our institution.

## Consent

Written informed consent was obtained from the patient for publication of this case report and accompanying images. A copy of the written consent is available for review by the Editor-in-Chief of this journal on request.

## Author contribution

Farzaneh Amini Nezhad participated in the study design and drafting the manuscript.

Moloud Payab participated in drafting the manuscript.

Sara Nayebandi participated in data collection.

Shirin Hasani-Ranjbar participated in the study design and interpretation.

All authors read, provided feedback and approved the final manuscript.

## Registration of research studies

NA.

## Guarantor

Dr. Shirin Hasani-Ranjbar

## Provenance and peer review

Not commissioned, externally peer-reviewed

## References

[1] Gouveia S. (2012). Persistent primary hyperparathyroidism: an uncommon location for an ectopic gland-case report and review. Arq. Bras. Endocrinol. Metabol..

[2] Piciu D. (2016). Primary hyperparathyroidism-Jaw tumor syndrome: a confusing and forgotten diagnosis. Clujul Med..

[3] Khan A., Bilezikian J. (2000). Primary hyperparathyroidism: pathophysiology and impact on bone. Can. Med. Assoc. J..

[4] Bandeira F. (2013). Diagnosis and management of primary hyperparathyroidism: a scientific statement from the Department of Bone Metabolism, the Brazilian Society for Endocrinology and Metabolism. Arq. Bras. Endocrinol. Metabol..

[5] Soltani A., Hasani-Ranjbar S., Moayyeri A. (2008). Hypocalcemia as a presentation for multifocal osteosarcoma. Pediatr. Blood Cancer.

[6] Bilezikian J.P. (2017). Hyperparathyroidism. Lancet.

[7] Belhoucha B. (2014). Simultaneous maxillary and mandibular brown tumors as the first presentation of secondary hyperparathyroidism. Am. J. Med. Case Rep..

[8] Shetty A.D., Namitha J., James L. (2015). Brown tumor of mandible in association with primary hyperparathyroidism: a case report. J. Int. Oral Health: JIOH.

[9] Triantafillidou K. (2006). Brown tumors of the jaws associated with primary or secondary hyperparathyroidism. A clinical study and review of the literature. Am. J. Otolaryngol..

[10] Udelsman R. (2011). Approach to the patient with persistent or recurrent primary hyperparathyroidism. J. Clin. Endocrinol. Metab..

[11] Agha R.A., Fowler A.J., Saetta A., Barai I., Rajmohan S., Orgill D.P., SCARE Group (2016). The SCARE statement: consensus-based surgical case report guidelines. Int. J. Surg..

[12] Chen Y. (2016). CDC73 gene mutations in sporadic ossifying fibroma of the jaws. Diagn. Pathol..

[13] Thakker R.V. (2016). Genetics of parathyroid tumours. J. Intern. Med..

[14] Kennedy R.A., Thavaraj S., Diaz-Cano S. (2017). An overview of autosomal dominant tumour syndromes with prominent features in the oral and maxillofacial region. Head Neck Pathol..

[15] du Preez H. (2016). Hyperparathyroidism jaw tumour syndrome: a pictoral review. Insights Imaging.

